# Domain swapping oligomerization of thermostable *c*-type cytochrome in *E. coli* cells

**DOI:** 10.1038/srep19334

**Published:** 2016-02-03

**Authors:** Yugo Hayashi, Masaru Yamanaka, Satoshi Nagao, Hirofumi Komori, Yoshiki Higuchi, Shun Hirota

**Affiliations:** 1 Graduate School of Materials Science, Nara Institute of Science and Technology, 8916-5 Takayama, Ikoma, Nara 630-0192, Japan.; 2 Faculty of Education, Kagawa University, 1-1 Saiwai, Takamatsu, Kagawa 760-8522, Japan.; 3 Department of Life Science, Graduate School of Life Science, University of Hyogo, 3-2-1 Koto, Kamigori-cho, Ako-gun, Hyogo 678-1297, Japan.; 4 RIKEN SPring-8 Center, 1-1-1 Koto, Sayo-cho, Sayo-gun, Hyogo 679-5148, Japan.

## Abstract

Knowledge on domain swapping *in vitro* is increasing, but domain swapping may not occur regularly *in vivo*, and its information in cells is limited. Herein, we show that domain-swapped oligomers of a thermostable *c*-type cytochrome, *Hydrogenobacter thermophilus* cyt *c*_552_, are formed in *E. coli* which expresses cyt *c*_552_. The region containing the N-terminal α-helix and heme was domain-swapped between protomers in the dimer formed in *E. coli*. The amount of cyt *c*_552_ oligomers increased in *E. coli* as the cyt *c*_552_ concentration was increased, whereas that of high-order oligomers decreased in the order of decrease in protein stability, indicating that domain swapping decreases in cells when the protein stability decreases. Apo cyt *c*_552_ was detected in the cyt *c*_552_ oligomer formed in *E. coli*, but not in that of the A5F/M11V/Y32F/Y41E/I76V mutant. The cyt *c*_552_ oligomer containing its apo protein may form at the periplasm, since the apo protein detected by mass measurements did not contain the signal peptide. These results show that domain-swapped cyt *c*_552_ oligomers were formed in *E. coli*, owing to the stability of the transient oligomer containing the apo protein before heme attachment. This is an indication that exceedingly stable proteins may have disadvantages forming domain-swapped oligomers in cells.

In domain swapping, a protein molecule exchanges its structural region with the corresponding region of another molecule of the same protein. Domain swapping was first reported by Eisenberg and co-workers for diphtheria toxin in 1994^1^. Since then, domain swapping has been reported for many proteins^2^,^3^,^4^. For example, ribonuclease (RNase) A swaps its N-terminal α-helix or C-terminal β-strand, forming two different domain-swapped structures^5^,^6^. The domain swapping of RNase A occurs during folding from its partially unfolded state^7^.

Domain swapping has also been reported for heme proteins^8^,^9^,^10^,^11^,^12^,^13^,^14^,^15^,^16^,^17^,^18^. We have shown that horse cytochrome (cyt) *c* domain swaps its C-terminal α-helix by treatment with ethanol^11^, or when refolding from its guanidinium ion-induced unfolded state^19^. The hydrophobic interaction between the N- and C-terminal α-helices at the early stage of folding is important for domain swapping in cyt *c*^19^. According to small angle X-ray scattering measurements, a certain amount of cyt *c* molecules interacted as oligomers in its molten globule state, whereas the domain-swapped dimer of cyt *c* was obtained by refolding from its molten globule state^20^. *Hydrogenobacter thermophilus* (HT) cyt *c*_552_ is a thermostable *c*-type cytochrome. Its high thermostability is achieved by the dense packing of the side chains of hydrophobic amino acids^21^,^22^,^23^,^24^,^25^. HT cyt *c*_552_ has been shown to form dimers by domain swapping, but the swapping region (N-terminal α-helix and heme) was different from that of horse cyt *c*^12^. The domain-swapped dimer of HT cyt *c*_552_ also exhibited high thermostability by maintaining the high density of the amino acid side chains as in the monomer, where the dimer did not dissociate to monomers at 70 °C^12^. For biosynthesis of HT cyt *c*_552_ and many gram-negative bacterial *c*-type cytochromes, the apo precursor protein is synthesized at the cytoplasm and transferred to the periplasm by a secretory protein^26^,^27^. After the signal peptide is cleaved at the periplasm, a heme is attached to the apo protein by a cyt *c* maturation (Ccm) system^28^. It is presumed that the Ccm system is a chaperone for the apo protein and avoids aggregation of the apo protein during the cyt *c* maturation process *in vivo*^29^.

Similarly, serine protease inhibitors antithrombin and α_1_-antitrypsin also form oligomers by domain swapping^30^,^31^. A disulfide variant of α_1_-antitrypsin has been shown to form oligomers in two model-cell systems, *Pichia pastoris* and COS-7 tissue culture cell, by trapping the domain-swapped structure^31^. When the C-terminal domain of severe acute respiratory syndrome coronavirus main protease (M^pro^-C) was expressed in *Escherichia coli* (*E. coli*), domain-swapped dimers were observed in addition to monomers^32^,^33^. The monomer-to-dimer ratio of M^pro^-C was three to two when purified from *E. coli*, whereas it was nine to one for the equilibrium at 37 °C *in vitro*^34^. Domain swapping has also induced two types of splicing when intein was expressed in *E. coli*^35^.

However, domain swapping may not occur regularly in cells, and the factors governing domain swapping *in vivo* remain unrevealed. In this study, we expressed thermostable HT cyt *c*_552_ in *E. coli* cells, and found that HT cyt *c*_552_ forms oligomers by domain swapping in *E. coli* cells. The amount of HT cyt *c*_552_ oligomers decreased when the protein stability was decreased. We attribute the decrease in oligomers to the decrease in stability of the transient complex containing the holo and apo proteins, during maturation of the apo protein to the holo protein by heme attachment.

## Results

### Oligomerization of HT cyt *c*_552_ in *E. coli* cells

**Figure 1:**
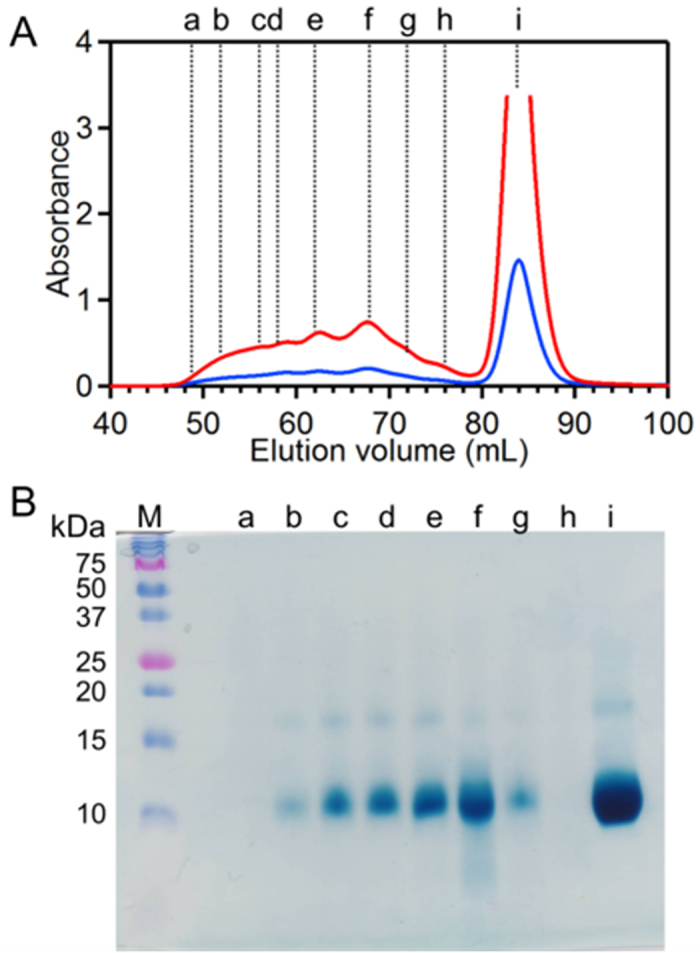
Size exclusion chromatogram of the HT cyt *c*_552_ solution obtained from *E. coli*, and SDS-PAGE analysis of the fractions: (**A**) Chromatogram and (**B**) heme-stained SDS-PAGE gel. Monitoring wavelengths for the chromatogram were 280 nm (blue) and 410 nm (red). Lanes in the SDS-PAGE gel: lane M, protein markers; lane a, 47–49 ml; lane b, 51–53 ml; lane c, 55–57 ml; lane d, 57–59 ml; lane e, 61–63 ml; lane f, 67–69 ml; lane g, 71–73 ml; lane h, 75–77 ml; lane i, 83–85 ml (The volumes represent the fraction positions in the chromatogram).

To investigate formation of oligomers in *E. coli*, we freeze-thawed *E. coli* cells expressing wild-type (WT) HT cyt *c*_552_, and performed size exclusion chromatography for the extracted solution. Peaks were observed at elution volumes of 50–75 and 85 ml in the chromatogram (Fig. 1A). The peak at 85 ml corresponded to that of monomeric HT cyt *c*_552_. The ratio of the absorption at 410 nm to that at 280 nm (Abs_410_/Abs_280_) for the fractions at 50–75 ml was about 3.6 (Fig. 1A). Heme staining was performed for the sodium dodecylsulfate (SDS)-PAGE gel of the fractions obtained by size exclusion chromatography to investigate the presence of heme *c* (Fig. 1B). A main band with a mass corresponding to the molecular weight of HT cyt *c*_552_ (9.2 kDa) was detected in all of the fractions at 50–75 ml. This value corresponded well to that of HT cyt *c*_552_. Therefore, we attributed the peak at 50–75 ml to HT cyt *c*_552_ oligomers. However, no oligomers were detected in the chromatogram when HT cyt *c*_552_ and cyt *c*_552_ free *E. coli* lysate were freeze-thawed together (Supplementary information, Fig. S1), indicating that HT cyt *c*_552_ oligomers were formed in *E. coli* by non-covalent association of its monomers.

### Structure of dimeric HT cyt *c*_552_ obtained from *E. coli*

**Figure 2:**
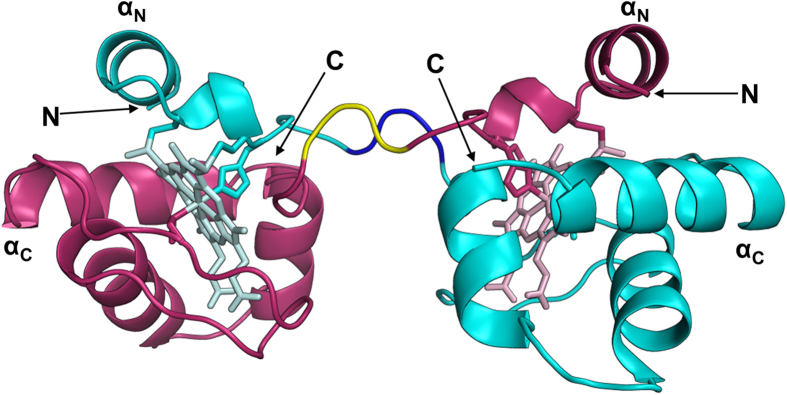
Crystal structure of dimeric HT cyt *c*_552_ obtained from *E. coli* (PDB ID: 4ZID). Each protomer is shown in magenta and cyan. The hemes and the side-chain atoms of His14 and Met59 are shown as stick models. The hinge loop (Ala18Lys19Lys20) is depicted in yellow and blue. The N- and C-termini and the N- and C-terminal helices are labeled as N, C, α_N_, and α_C_, respectively.

To elucidate the detailed structure of dimeric HT cyt *c*_552_ obtained from the *E. coli* expression system, we purified dimeric HT cyt *c*_552_ and solved its X-ray crystallographic structure (PDB code: 4ZID) (Supplementary information, Table S1). There was one HT cyt *c*_552_ protomer in the asymmetric unit of the crystal, and a domain-swapped structure of dimeric HT cyt *c*_552_ was obtained at 1.8 Å resolution (Fig. 2). The hinge loop was composed of Ala18, Lys19, and Lys20. The N-terminal α-helix from Asn1 to Lys17, together with the heme, was swapped between protomers in the dimer. The dimeric structure was similar to that obtained previously by treatment with ethanol (PDB code: 3VYM)^12^. The root-mean-square deviation value of Cα carbons between the protomer structure of the dimer obtained from the *E. coli* expression system and that obtained previously by treatment with ethanol (PDB code: 3VYM) was 0.43 Å, indicating that the protomer structures were similar.

### Effect of expression amount of HT cyt *c*_552_ on oligomer formation

**Figure 3:**
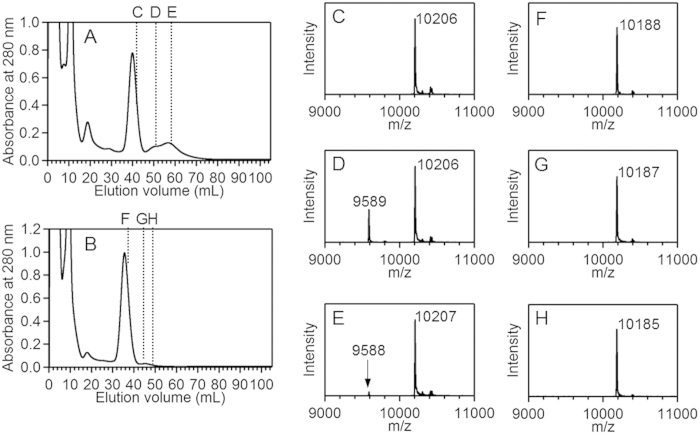
Ni affinity chromatograms of the HT cyt *c*_552_ solution extracted from *E. coli*, and MALDI-TOF mass spectra of the fractions. Chromatograms of (**A**) WT and (**B**) A5F/M11V/Y32F/Y41E/I76V HT cyt *c*_552_ solutions, and mass spectra of the fractions at (**C**) 42-44 ml, (**D**) 50-52 ml, and (**E**) 58-60 ml for WT HT cyt *c*_552_ and (**F**) 36-38 ml, (**G**) 44-46 ml, and (H) 48-50 ml for A5F/M11V/Y32F/Y41E/I76V HT cyt *c*_552_ are shown.

A His-tag (GSGHHHHHH) was attached to the C-terminus of WT HT cyt *c*_552_ to simplify the purification and analyze oligomer formation by Ni affinity chromatography using a His-trap column. The WT holo monomer was detected at ~40 ml in the Ni affinity chromatogram, whereas WT holo dimer and trimer were detected at ~48 and ~53 ml, respectively (Supplementary information, Fig. S2). By performing Ni affinity chromatography for the protein solution obtained from the *E. coli* cells expressing the WT protein and A5F/M11V/Y32F/Y41E/I76V (quintuple) mutant, red solutions were eluted at 36-75 ml and 32–50 ml, respectively (Fig. 3A,B).

**Figure 4:**
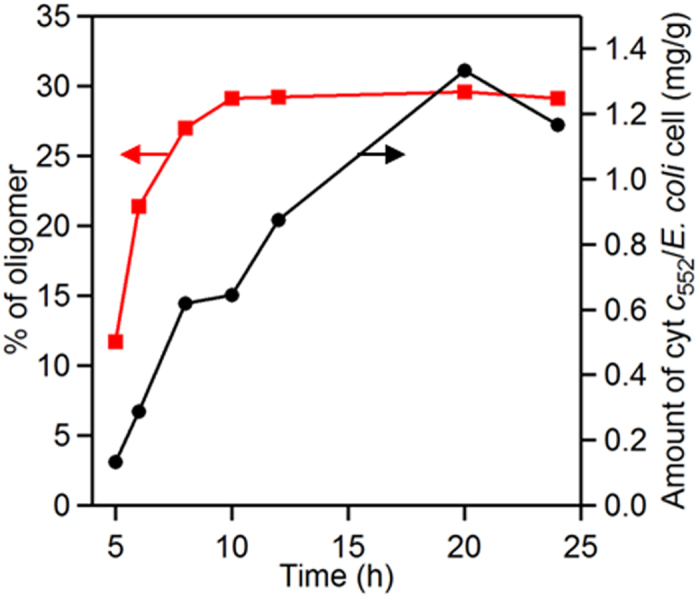
HT cyt *c*_552_ oligomer percentage (red) and HT cyt *c*_552_ expression amount per 1 g of *E. coli* (black) at various culturing times.

The HT cyt *c*_552_ expression amount per 1 g of *E. coli* cell increased rapidly with longer culturing time from 5 to 12 h, and gradually after 12 h (Fig. 4). The HT cyt *c*_552_ oligomer (dimer and higher order oligomers) amount also increased with longer culturing time from 5 to 12 h (Fig. 4). These results showed that HT cyt *c*_552_ oligomers increased as the HT cyt *c*_552_ concentration increased in *E. coli* cells.

### Effect of stability of HT cyt *c*_552_ on oligomer formation

To elucidate the effect of protein stability on oligomer formation in *E. coli* cells, we investigated oligomerization of His-tag-attached WT HT cyt *c*_552_ together with I76V, A5F/M11V, Y32F/Y41E, and quintuple mutant HT cyt *c*_552_, mutating amino acids important for protein stability. The order of protein stability of WT, I76V, A5F/M11V, Y32F/Y41E, and quintuple mutant HT cyt *c*_552_ (WT > I76V > A5F/M11V > Y32F/Y41E > quintuple mutant) has been elucidated by measuring the denaturing temperature and guanidine hydrochloride denaturing concentration (WT, 121.1 °C and 4.46 M; I76V, 117.8 °C and 3.98 M; A5F/M11V, 117.0 °C and 3.70 M; Y32F/Y41E, 108.3 °C and 2.69 M; quintuple mutant, 95.2 °C and 1.67 M)^36^. The expression amount of the cyt *c* protein per 1 g of *E. coli* cell was 1.5 ± 0.2, 1.8 ± 0.2, 0.7 ± 0.1, 1.8 ± 0.3, and 2.0 ± 0.2 mg/g for WT, I76V, A5F/M11V, Y32F/Y41E, and quintuple mutant HT cyt *c*_552_, respectively.

A peak was observed at m/z = 10,206 in the MALDI-TOF mass spectrum of the 42-44-ml fraction (shoulder of the 40-ml peak) obtained by Ni affinity chromatography of the WT HT cyt *c*_552_ (Fig. 3C). This mass corresponded well to the molecular weight of the WT holo protein (Mw = 10,205). The peak position at 50-60 ml in the Ni affinity chromatogram of the WT protein corresponded well to those of the oligomers larger than the dimer (Fig. 3A and Supplementary information, Fig. S2). A peak with a mass corresponding well to the molecular weight of the WT holo protein was also detected in the mass spectra of the fractions at 50-52 ml and 58-60 ml (Fig. 3D,E), indicating that the peak at 50-60 ml is attributable to oligomers constructed by WT monomeric cyt *c*_552_.

**Figure 5:**
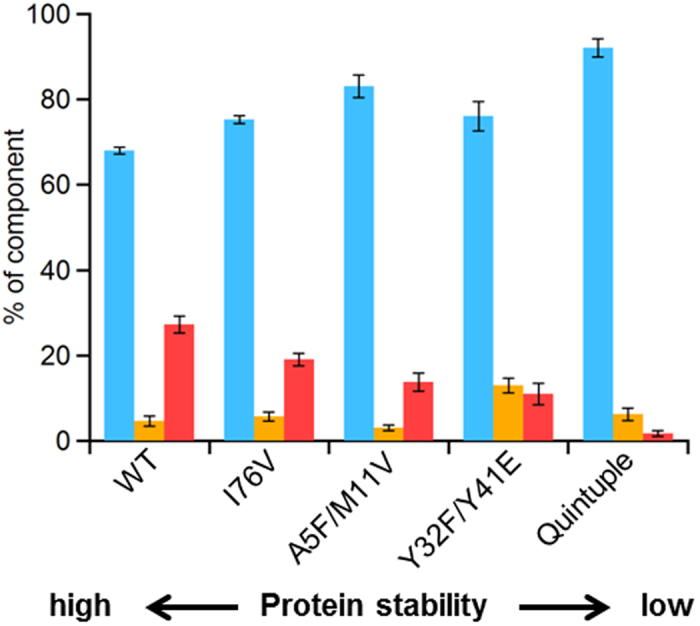
Percentages of monomer, dimer, and high-order oligomers (larger than dimer) obtained from *E. coli* cells expressing WT and mutant HT cyt *c*_552_. Monomer, dimer, and high-order oligomers are shown in blue, yellow, and red bars, respectively. The order of protein stability is shown at the bottom.

In the Ni affinity chromatograms of the protein solution obtained from the *E. coli* cells expressing WT, I76V, or A5F/M11V HT cyt *c*_552_, relatively narrow and broad peaks were observed at ~40 ml and 45-75 ml, respectively, corresponding to the monomer and oligomers, respectively (Supplementary information, Fig. S3). Peaks were observed at ~40 ml and ~48 ml corresponding to the monomer and dimer, respectively, in the Ni affinity chromatograms obtained from the *E. coli* cells expressing Y32F/Y41E and quintuple mutants (Supplementary information, Fig. S3), although there was no significant difference among growth of *E. coli* cells expressing WT and mutant proteins. The monomer, dimer, and higher-order oligomer (higher than dimer) amounts were estimated from the peak areas in the chromatograms of WT and mutant proteins (Fig. 5 and Supplementary information, Fig. S3). The amount of high-order oligomers decreased in the order of WT > I76V > A5F/M11V > Y32F/Y41E > quintuple mutant, corresponding to the decrease in protein stability. Taking the results into consideration, we propose that the oligomer amount of HT cyt *c*_552_ decreases in *E. coli* when the protein stability decreases.

### Detection of HT apo cyt *c*_552_ in oligomers

An additional peak from that of the WT holo protein was observed at m/z = 9,588–9,589 in the mass spectra of the fractions at 50-52 ml and 58-60 ml in the Ni affinity chromatogram of the WT protein (Fig. 3D,E). The mass of the additional peak corresponded well to the molecular weight of the protonated WT apo protein with a His-tag attached and the cysteine residues oxidized (Mw = 9,590). These results indicated that HT cyt *c*_552_ oligomers containing the apo protein were produced in *E. coli*. No peak corresponding to the mass of His-tag-attached apo protein was detected in the mass spectrum of the fraction at 42-44 ml (Fig. 3C), although WT apo monomer was eluted at ~41 ml by Ni affinity chromatography (Supplementary information, Fig. S2). No apo cyt *c*_552_ was obtained as a monomer by purification from *E. coli* cells, presumably because the apo monomer decomposed relatively easily in the cells.

The intensity of the oligomer peaks decreased significantly in the Ni affinity chromatogram for the solution obtained from *E. coli* expressing the His-tag-attached quintuple mutant (Fig. 3B). In the mass spectra of the fractions at 36-38, 44-46, and 48-50 ml obtained by the Ni affinity chromatography of the quintuple mutant, a peak with a mass corresponding well to the molecular weight of the quintuple holo mutant (Mw = 10,189) was detected, whereas no peak corresponding to the mass of its apo protein was detected (Fig. 3F–H). The decrease in the amount of oligomers containing the apo protein may result in a decrease in formation of domain-swapped oligomers. However, no peak corresponding to the mass of the apo protein with the signal peptide was detected in the mass spectra of WT protein and quintuple mutant, giving evidence that apo cyt *c*_552_ may co-exist with the holo protein at the periplasm of *E. coli*.

### Interaction of HT holo and apo cyt *c*_552_ during folding

To investigate the effect of the apo protein on the holo protein folding, the His-tag-attached holo protein was unfolded by an addition of guanidinium ion and refolded *in vitro* in the absence and presence of the apo protein using a desalting column^19^. After refolding the holo protein, the solution was subjected to Ni affinity chromatography. Peaks were observed at ~44 and 50–62 ml in the Ni affinity chromatograms of the solution obtained by folding in the absence and presence of the apo protein (Supplementary information, Fig. S4A). Precipitation was observed by refolding the holo protein in the presence of the apo protein, but not in the absence of it. The precipitate was colorless, indicating that some amount of the apo protein precipitated during folding. Therefore, the 280-nm absorbance in the Ni affinity chromatogram for the solution containing the apo protein was not twice as high as that without the apo protein. The 410-nm absorption at ~59 ml in the Ni affinity chromatogram increased by performing folding in the presence of the apo protein compared to that in the absence of it, indicating that the oligomers containing the holo protein increased by the addition of the apo protein during folding. Peaks corresponding to the mass of the apo protein were observed at m/z = 9,589 and 9,586 in the MALDI-TOF mass spectra of the fractions at 54-56 and 58-60 ml, respectively (Supplementary information, Fig. S4C,D), showing that oligomers containing the apo protein were produced by refolding the holo protein in the presence of the apo protein. For His-tag-attached quintuple mutant HT holo cyt *c*_552_, peaks were observed at about ~43 and ~54 ml in the Ni affinity chromatograms of the solution refolded from the guanidinium ion-induced unfolded state in the absence and presence of the apo protein (Supplementary information, Fig. S4B). The difference was small between the elution curves obtained by refolding the quintuple mutant in the absence and presence of the apo protein, and no peak corresponding to the mass of the apo protein was observed in the mass spectra of the oligomer fractions at 52-54 and 56-58 ml obtained by the Ni affinity chromatography (Supplementary information, Fig. S4E,F). These results show that no oligomers containing the apo protein were detected by refolding the holo and apo quintuple mutant together *in vitro*, although oligomers were constructed with only the holo protein.

## Discussion

A monomeric protein may oligomerize or its oligomer may dissociate to monomers when extracting the protein from living cells. Therefore, careful treatment is necessary to investigate oligomerization of proteins in cells. HT cyt *c*_552_ is a stable protein^36^,^37^, and it hardly denatures and forms oligomers after it folds in a native structure. Oligomers of HT cyt *c*_552_ were detected when it was expressed in *E. coli* (Fig. 1), and the amount of oligomers increased when the amount of HT cyt *c*_552_ expressed was increased (Fig. 4). These results clearly showed that HT cyt *c*_552_ oligomerizes by intermolecular interaction in *E. coli* cells.

Intermolecular interaction at the initial stage of folding is important for formation of domain-swapped oligomers in horse cyt *c* and horse myoglobin^19^,^38^. However, HT cyt *c*_552_ molecules fold simultaneously *in vitro*, and thus folding *in vitro* and that in *E. coli* cells are different. A heme is attached to apo HT cyt *c*_552_ at the periplasm by the Ccm system consisted of eight proteins (CcmA–CcmH)^39^. The lifetime of *Bradyrhizobium japonicum* apo *c*-type protein without a signal peptide is estimated to be 2–5 min in the periplasm of *E. coli*^40^. Therefore, an apo *c*-type protein may co-exist with its holo *c*-type protein during maturation in cells.

Oligomers containing the apo protein were detected when His-tag-attached WT HT cyt *c*_552_ was purified from *E. coli* (Fig. 3). The amount of high-order oligomers against monomers in *E. coli* decreased as the protein stability was decreased (Fig. 5). In addition, the apo protein was not detected in the mass spectra of the fractions of the oligomers in the Ni affinity chromatogram of the quintuple mutant (Fig. 3F–H). For refolding *in vitro*, the WT holo protein formed oligomers with its apo protein, whereas the quintuple mutant did not (Supplementary information, Fig. S4). The α-helical content of the apo protein of the WT protein and quintuple mutant was estimated at 19% and 11%, respectively, according to the circular dichroism (CD) spectra, whereas that of the holo protein was estimated at 49% and 42%, respectively (Supplementary information, Fig. S5). These results showed that the holo and apo proteins of the quintuple mutant were unfolded more compared to the corresponding forms of the WT protein. The stability of the mutant oligomer containing the apo form may decrease compared to that of the WT protein oligomer due to decrease in protein stability, causing decrease in formation of the domain-swapped oligomers in *E. coli*.

**Figure 6:**
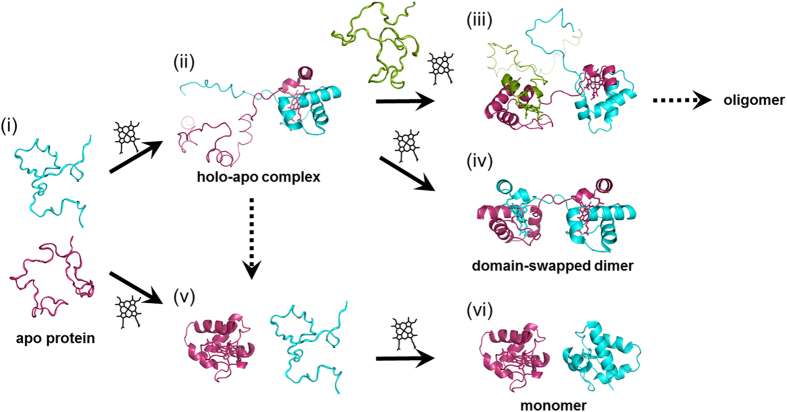
Schematic view of oligomer formation of HT cyt *c*_552_ in *E. coli*. (i) Model of unfolded apo protein. (ii) Model of transient holo-apo complex. (iii) Model of high order holo-apo complex. (iv) Dimeric holo protein (PDB ID: 4ZID). (v) Monomeric holo protein (PDB ID: 1YNR) and model of apo protein. (vi) Monomeric holo proteins (PDB ID: 1YNR). Different HT cyt *c*_552_ molecules are shown in magenta, cyan, and green. Hemes are shown as black stick models.

Based on the present results, we propose an oligomerization process for HT cyt *c*_552_ in cells (Fig. 6). When a holo protein is formed, it creates a transient oligomer with an apo protein. A heme is inserted to the apo protein of the transient holo-apo complex, refolding to a domain-swapped dimer. Higher order oligomers may form when an apo protein interacts with two holo proteins. For less stable proteins, the holo-apo complex dissociates to holo and apo monomers, eventually forming only holo monomers. Although apo cyt *c* has been shown to decompose by DegP protease at the periplasm of *E. coli*^41^, the apo protein of thermostable HT cyt *c*_552_ survives from decomposition in the cells by forming a transient complex with its holo proteins. Our results reveal that more domain-swapped oligomers are formed for more stable proteins, and exceedingly stable proteins may have disadvantages by forming oligomer by-products in cells.

## Methods

### Construction of HT cyt *c*_552_ expression system

The pKO2 plasmid DNA coding WT HT cyt *c*_552_, its modified plasmid DNA coding I76V, A5F/M11V, Y32F/Y41E, or A5F/M11V/Y32F/Y41E/I76V HT cyt *c*_552_, and pEC86 plasmid DNA coding the Ccm system with CcmA–CcmH were provided by Prof. Yoshihiro Sambongi (Hiroshima Univeristy)^36^. Insertion of a His-tag (GSGHHHHHH) to HT cyt *c*_552_ was performed by PCR-based *in vitro* mutagenesis. A more complete description of the procedures can be found in supplementary information.

### Culture of *E. coli* and purification of HT cyt *c*_552_

HT cyt *c*_552_ was expressed in *E. coli* JCB387. All growths were performed aerobically in LB medium at 37 °C. For analysis of HT cyt *c*_552_ expression, a partial amount of *E. coli* cells was collected by centrifugation (8,000 g, 5 min, 4 °C) after culturing the cells for 5, 6, 8, 10, 12, 20, and 24 h, and subsequently weighed. The SDS-PAGE gel was heme-stained for detection of heme *c*^42^. WT HT cyt *c*_552_ without a His-tag was purified as reported previously^12^. His-tag-attached WT, I76V, A5F/M11V, Y32F/Y41E, and quintuple mutant HT cyt *c*_552_ were extracted from *E. coli* by freeze-thaw and sonication, and purified with a Ni affinity column (HisTrap HP, GE Healthcare). A more complete description of the procedures can be found in supplementary information.

### Preparation of HT apo cyt *c*_552_

WT and quintuple mutant HT apo cyt *c*_552_ were prepared according to the published method^43^,^44^. A detailed description of the procedures can be found in supplementary information.

### X-ray crystallographic analysis

Dimeric HT cyt *c*_552_ obtained from *E. coli* by freeze-thaw was purified as reported^12^. Crystallization of dimeric HT cyt *c*_552_ without a His-tag was performed at room temperature using the sitting-drop vapor-diffusion method with a crystal plate (CrystalClear D Strips, Douglas Instruments, Hampton Research, CA). The diffraction data were collected at the BL38B1 beamline at SPring-8, Japan, using a Quantum315 detector (ADSC). Additional details on the experimental procedures are provided in supplementary information.

### Spectroscopic measurements

CD spectra of WT and A5F/M11V/Y32F/Y41E/I76V HT holo and apo cyt *c*_552_ without a His-tag were measured with a J-725 CD spectropolarimeter (Jasco, Japan) using a 0.1-cm-path-length quartz cell at 25 °C. MALDI-TOF mass spectra of HT cyt *c*_552_ were obtained with an Autoflex II mass spectrometer (Bruker Daltonics) using sinapinic acid as a matrix in linear mode. Additional details on the experimental procedures are provided in supplementary information.

### Refolding of HT cyt *c*_552_

Refolding of His-tag-attached HT cyt *c*_552_ was performed using a desalting gel column (PD SpinTrap G-25, GE Healthcare) at 4 °C as reported previously^19^. Additional details on the experimental procedures are provided in supplementary information.

## Additional Information

**How to cite this article**: Hayashi, Y. *et al.* Domain swapping oligomerization of thermostable *c*-type cytochrome in *E. coli* cells. *Sci. Rep.* **6**, 19334; doi: 10.1038/srep19334 (2016).

## Supplementary Material

Supplementary Information
