# Supplementary material for: Domain swapping oligomerization of thermostable c-type cytochrome in E. coli cells
Source: Sci Rep. 2016 Feb 3;6:19334. doi: 10.1038/srep19334 (PMC4738263; doi:10.1038/srep19334)
Supplement: Supplementary Information [file srep19334-s1.pdf]

Supplementary Information

Domain swapping oligomerization of thermostable *c*-type cytochrome in *E. coli* cells

Yugo Hayashi[1], Masaru Yamanaka[1], Satoshi Nagao[1], Hirofumi Komori[2], Yoshiki Higuchi[3,4], and Shun Hirota[1]

[1]Graduate School of Materials Science, Nara Institute of Science and Technology, 8916-5 Takayama, Ikoma, Nara 630-0192, Japan. [2]Faculty of Education, Kagawa University, 1-1 Saiwai, Takamatsu, Kagawa 760-8522, Japan. [3]Department of Life Science, Graduate School of Life Science, University of Hyogo, 3-2-1 Koto, Kamigori-cho, Ako-gun, Hyogo 678-1297, Japan. [4]RIKEN SPring-8 Center, 1-1-1 Koto, Sayo-cho, Sayo-gun, Hyogo 679-5148, Japan.

Correspondence and requests for materials should be addressed to S.H. (email: hirota@ms.naist.jp)

Materials and Methods

Construction of HT cyt c_{552} expression system. Insertion of a His-tag (GSGHHHHHH) at the C-termini of WT I76V, A5F/M11V, Y32F/Y41E, and A5F/M11V/Y32F/Y41E/I76V HT cyt c_{552} was performed by PCR-based *in vitro* mutagenesis of the plasmid DNA coding HT cyt c_{552} with a KOD Plus mutagenesis kit (TOYOBO) using forward (GGCTCGGGCCATCATCATCATCATCATTAAGTCGACCTGCAGCCAAGCTT) and reverse primers (CTTTATGGAGAGTATCCACTGGGC for WT, A5F/M11V, and Y32F/Y41E HT cyt c_{552}; CTTTATGGAGAGTACCCACTGGGC for I76V and A5F/M11V/Y32F/Y41E/I76V HT cyt c_{552}). The plasmid DNAs were enhanced with DH5α cells, and transformed into *E. coli* JCB387 containing the pEC86 plasmid DNA[1].

Purification of HT cyt c_{552}. The cultured *E. coli* cells were suspended with 25 mM acetic acid buffer, pH 5.0, and centrifuged (13,700 g, 10 min, 4 °C). The cells were suspended again with 100 mM Tris-HCl buffer, pH 8.0, containing 10 mM EDTA and 20% (w/v) sucrose, and subsequently incubated on ice for 1 h and centrifuged (13,700 g, 10 min, 4 °C). The cells were finally suspended with 100 mM potassium phosphate buffer, pH 7.0, freeze-thawed, and centrifuged (13,700 g, 10 min, 4 °C). Streptomycin (1% w/v, Nacalai tesque) was added to the supernatant, and the solution was incubated on ice for 30 min and subsequently centrifuged (13,700 g, 10 min, 4 °C). The solution was dialyzed overnight at 4 °C with 10 mM potassium phosphate buffer, pH 7.0, containing 1 mM streptomycin. Oxidized HT cyt c_{552} was prepared by an addition of 50 mM potassium ferricyanide to the sample solution (final concentration, 50 μM), and purified with a CM Sepharose (Wako) column. The elution solution containing oligomeric HT cyt c_{552} was analyzed by size exclusion chromatography (column: Hiload 16/600 Superdex75 pg or Superdex 75 10/300 GL, GE Healthcare) using a FPLC system (BioLogic DuoFlow 10, Bio-Rad) as reported[2]. SDS-PAGE was performed with 18% gel for the fractions obtained by size exclusion chromatography. The gels were heme-stained with 3,3',5,5'-tetramethylbenzidine (TCI, Tokyo, Japan) and hydrogen peroxide for detection of heme *c*[3].

WT and A5F/M11V/Y32F/Y41E/I76V HT cyt c_{552} without a His-tag were purified with a CM Sepharose (Wako) column and size exclusion chromatography (column: Hiload 26/600 Superdex75 pg, GE Healthcare) using the FPLC system (BioLogic DuoFlow 10, Bio-Rad) as reported[2]. His-tag-attached WT, I76V, A5F/M11V, Y32F/Y41E, and A5F/M11V/Y32F/Y41E/I76V HT cyt c_{552} were extracted from *E. coli* by freeze-thaw and sonication. The amount of extracted His-tag-attached HT cyt c_{552} was estimated by the pyridine hemochrome method[4]. The His-tag-attached HT cyt c_{552} were purified with a HisTrap HP column (GE Healthcare) using the FPLC system (BioLogic DuoFlow 10, Bio-Rad) (flow rate, 1.0 mL/min; monitoring wavelength, 280 nm and 410 nm; gradient, 25 mM Tris-HCl buffer, pH 8.0, containing 0.5 M NaCl and the same buffer containing 0.5 M NaCl and 0.5 M imidazole; temperature, 4 °C). *E. coli* JCB387 not containing pKO2 and pEC86 plasmid DNAs was cultured to investigate the effect of freeze-thaw on oligomerization.

Preparation of HT apo cyt c_{552}. After reaction of HT cyt c_{552} with 10 mg/ml Ag_2SO_4, the solution containing the apo protein was treated with 1 M dithiothreitol and 6 M guanidine hydrochloride. The obtained solution was incubated at 25 °C under dark for 4 h, and subsequently centrifuged (20,000 g, 15 min, 20 °C). The supernatant was dialyzed overnight with 25 mM sodium acetate buffer, pH 5.0, and 25 mM Tris-HCl buffer, pH 8.0, for HT apo cyt c_{552} without a His-tag and His-tag-attached HT apo cyt c_{552}, respectively. HT apo cyt c_{552} without a His-tag and His-tag-attached HT apo cyt c_{552} were subsequently purified with HiTrap SP and HisTrap HP columns (GE Healthcare), respectively, using the FPLC system (BioLogic DuoFlow 10, Bio-Rad). The two cysteine residues which were originally bound to the heme formed an intramolecular disulfide bond. Oxidation of the cysteine residues of HT apo cyt c_{552} was confirmed by Ellman's regent[5].

X-ray crystallographic analysis. Oxidized dimeric HT cyt c_{552} without a His-tag was dissolved in 100 mM HEPES buffer, pH 7.0, at a protein concentration of 3.0 mM (heme unit). Droplets prepared by mixing 2 μL of the protein solution with 2 μL reservoir solution were equilibrated. The reservoir solution was 1.6 M sodium citrate buffer, pH 6.5. A crystal was observed in the protein solution after incubation at room temperature for three days.

The crystal was mounted on a cryo-loop without an additional cryoprotectant, and flash-frozen at 100 K in a nitrogen cryo system. The crystal-to-detector distance was 250.0 mm, and the wavelength was 1.0 Å. The oscillation angle was 1°, and the exposure time was 12.0 s per frame. The total number of frames was 180. The diffraction data were processed using the program, HKL2000[6]. The preliminary structure was obtained by a molecular replacement method (MOLREP)[7] using the atomic coordinates of the structure of dimeric HT cyt c_{555} (PDB code: 3VYM) as a starting model. The structure refinement was performed using the program, REFMAC[8]. The molecular model was manually corrected, and water molecules were picked up in the electron density map using the program, COOT[9]. The data collection and refinement statistics are summarized in Table SI (Supporting Information). The three-dimensional structure of the protomer of the dimer obtained from the *E. coli* expression system and that obtained by ethanol treatment were compared using the molecular graphics program, PyMOL[10].

Spectroscopic measurements. The concentrations of WT and A5F/M11V/Y32F/Y41E/I76V HT holo cyt c_{552} without a His-tag were calculated from the absorbance of the Soret band at 410 nm using the coefficient reported for WT HT cyt c_{552} (ε_{410}= 109,000 $M^{-1}cm^{-1}$)[2] and that obtained for A5F/M11V/Y32F/Y41E/I76V HT cyt c_{552} by the pyridine hemochrome method (ε_{410} = 112,000 $M^{-1}cm^{-1}$)[4]. The concentration of HT apo cyt c_{552} without a His-tag was calculated from the absorbance at 280 nm using the coefficients obtained by using the program, ProtParam tool, on the ExPASy server (WT, ε_{280} = 15,600 $M^{-1}cm^{-1}$; A5F/M11V/Y32F/Y41E/I76V, ε_{280} = 12,600 $M^{-1}cm^{-1}$)[11]. Concentration of His-tag-attached proteins were calculated using the absorption coefficients of the proteins without a His-tag.

The α-helical content was estimated from the Cotton effect at 222 nm by the equation previously reported[12].

$$f_H = \frac{-[\theta]_{222} - 2340}{30300}$$

The buffer of the sample was exchanged with ultrapure water using an Amicon ultrafiltration tube (Merck Millipore) before mass measurements.

Refolding of HT cyt c_{552}. Oxidized His-tag-attached WT and A5F/M11V/Y32F/Y41E/I76V HT holo cyt c_{552} (0.3 mM) in the unfolded state with and without its apo protein (0.3 mM) were obtained by an addition of guanidine hydrochloride (final concentration, 5.33 M) in 50 mM potassium phosphate buffer, pH 7.0. After the HT cyt c_{552} solution was passed through the desalting gel column, the solution was incubated at 37 °C for 1h. The fractions were analyzed by Ni affinity chromatography (column: HisTrap HP, GE Healthcare) using the FPLC system (BioLogic DuoFlow 10, Bio-Rad) and MALDI-TOF mass spectroscopy.

Table S1. Statistics of data collection and structure refinement.

Data collection	
X-ray source	SPring-8 (BL38B1)
Wavelength (Å)	1.0000
Space group	$P3_121$
Unit cell parameters	
a, *b*, *c* (Å)	45.8, 45.8, 78.3
α, β, γ (°)	90.0, 90.0, 120
Resolution (Å)	50.00−1.80 (1.86−1.80)
Number of unique reflections	9242 (847)
R_{merge}[a]	0.070 (0.649)
Completeness (%)	99.2 (93.9)
$<I/\sigma(I)>$	37.5 (2.2)
Redundancy	9.7(7.1)
Refinement	
Resolution (Å)	39.67−1.80 (1.85−1.80)
Number of reflections	8721 (616)
R_{work}[b]	0.167 (0.256)
R_{free}[b]	0.203 (0.292)
Completeness (%)	98.7 (91.9)
Number of atoms in an asymmetric unit	
Protein	601
Water	29
Heme	43
Average *B* factors (Å^2)	
Protein	39.4
Water	40.4
Heme	26.0
Ramachandran plot (%)	
Favored	100
Allowed	0
Outlier	0

Statistics for the highest-resolution shell are given in parentheses.

[a] $R_{merge}=\Sigma_{hkl} \mid I - <I> \mid (\Sigma_{hkl} \mid I \mid)^{-1}$.

[b] $R_{work}=\Sigma_{hkl} \mid \mid F_{obs} \mid - k \mid F_{calc} \mid \mid (\Sigma_{hkl} \mid F_{obs} \mid)^{-1}$, *k*: scaling factor. R_{free} was computed identically, except where all reflections belong to a test set of 5 % of randomly selected data.

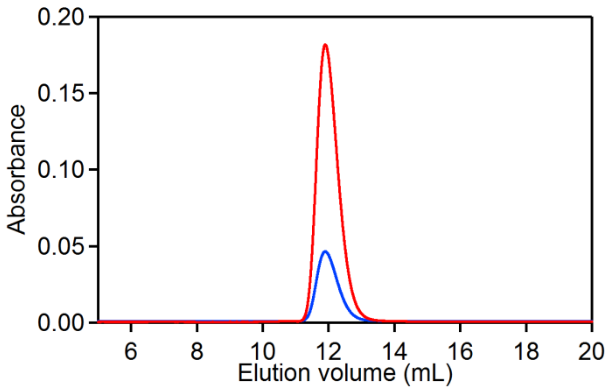

Fig. S1. Elution curves of the solution after freeze-thaw of the mixture of HT cyt c_{552} and cell lysate of *E. coli* JCB387. Measurement conditions: column, Superdex 75 10/300 GL; flow rate, 0.5 ml/min; monitoring wavelength, 280 nm (blue) and 410 nm (red); solvent, 50 mM potassium phosphate buffer, pH 7.0; temperature, 4 °C.

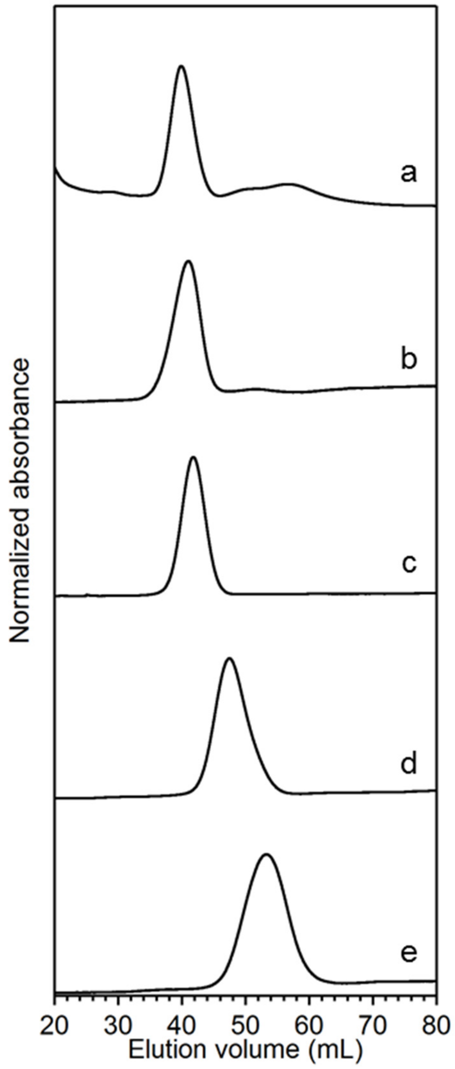

Fig. S2. Ni affinity chromatograms of (a) HT cyt c_{552} solution extracted from *E. coli*, (b) apo monomer, (c) holo monomer, (d) holo dimer, and (e) holo trimer. Measurement conditions: column, HisTrap HP; flow rate, 1.0 ml/min; monitoring wavelength, 280 nm; solvent, 25 mM Tris-HCl, pH 8.0, containing 0.5 M NaCl; imidazole gradient, 0.02–0.5 M; temperature, 4 °C.

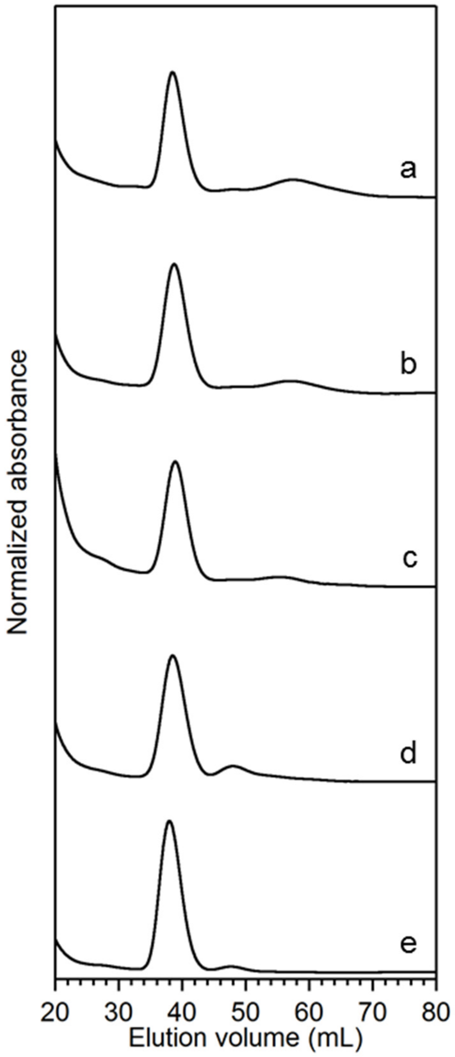

Fig. S3. Ni affinity chromatograms of (a) WT, (b) I76V, (c) A5F/M11V, (d) Y32F/Y41E, and (e) A5F/M11V/Y32F/Y41E/I76V HT cyt c_{552} solutions extracted from *E. coli.* Measurement conditions: column, HisTrap HP; flow rate, 1.0 ml/min; monitoring wavelength, 280 nm; solvent, 25 mM Tris-HCl, pH 8.0, containing 0.5 M NaCl; imidazole gradient, 0.02–0.5 M; temperature, 4 °C.

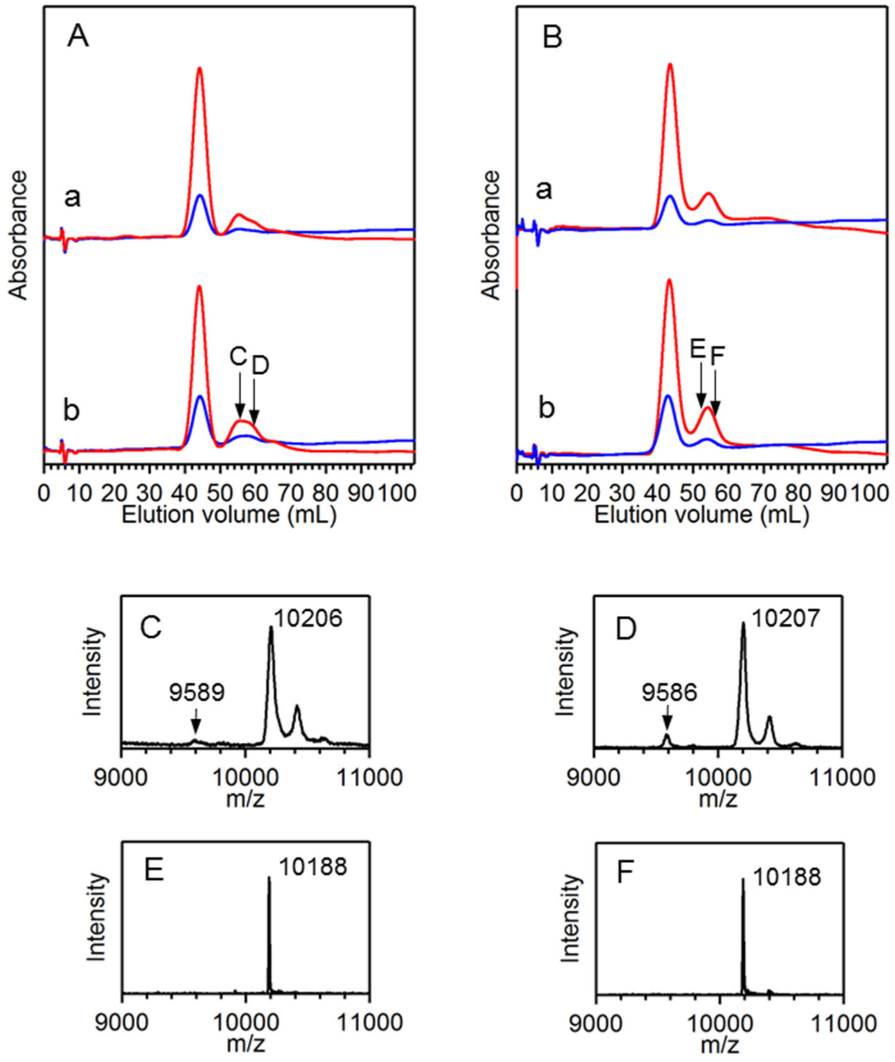

Fig. S4. Ni affinity chromatograms of the solution after refolding oxidized HT holo cyt c_{552} (0.3 mM) and MALDI-TOF mass spectra of the fractions. Chromatograms of (A) WT and (B) A5F/M11V/Y32F/Y41E/I76V HT holo cyt c_{552} (0.3 mM) after refolding in the (a) presence and (b) absence of the corresponding apo protein (0.3 mM) are shown. HT cyt c_{552} was refolded with the desalting method from the unfolded state in 50 mM potassium phosphate buffer, pH 7.0, containing 5.33 M GdnHCl at 4 °C. Mass spectra of the Ni affinity chromatography fractions at (C) 54–56 ml and (D) 58–60 ml for the WT protein and those at (E) 52–54 ml and (F) 56–58 ml for the A5F/M11V/Y32F/Y41E/I76V mutant are shown.

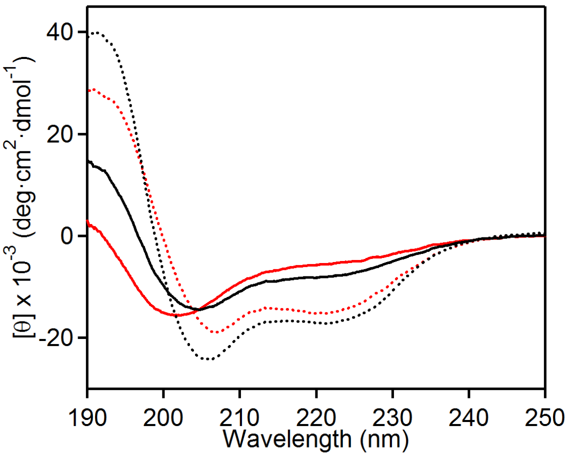

Fig. S5. CD spectra of oxidized WT (black) and A5F/M11V/Y32F/Y41E/I76V (red) HT holo (solid line) and apo (dotted line) cyt c_{552}. The protein concentration was calculated from the intensity of the Soret band for the holo proteins and the absorbance at 280 nm for the apo proteins. Measurement conditions: protein concentration, 9.5–10.5 μM; solvent, 50 mM potassium phosphate buffer, pH 7.0; temperature, 25 °C.

References

1. Arslan, E., Schulz, H., Zufferey, R., Künzler, P. & Thöny-Meyer, L. Overproduction of the *Bradyrhizobium japonicum c*-type cytochrome subunits of the *cbb*$_3$ oxidase in *Escherichia coli*. *Biochem. Biophys. Res. Commun.* **251**, 744-747 (1998).
2. Hayashi, Y. *et al.* Domain swapping of the heme and N-terminal α-helix in *Hydrogenobacter thermophilus* cytochrome *c*$_{552}$ dimer. *Biochemistry* **51**, 8608-8616 (2012).
3. Thomas, P.E., Ryan, D. & Levin, W. Improved staining procedure for detection of peroxidase-activity of cytochrome P450 on sodium dodecyl-sulfate polyacrylamide gels. *Anal. Biochem.* **75**, 168-176 (1976).
4. Berry, E.A. & Trumpower, B.L. Simultaneous determination of hemes *a*, *b,* and *c* from pyridine hemochrome spectra. *Anal. Biochem.* **161**, 1-15 (1987).
5. Riddles, P.W., Blakeley, R.L. & Zerner, B. Reassessment of Ellman reagent. *Methods Enzymol.* **91**, 49-60 (1983).
6. Otwinowski, Z. & Minor, W. Processing of X-ray diffraction data collected in oscillation mode. *Methods Enzymol.* **276**, 307-326 (1997).
7. Vagin, A. & Teplyakov, A. MOLREP: an automated program for molecular replacement. *J. Appl. Crystallogr.* **30**, 1022-1025 (1997).
8. Murshudov, G.N. *et al.* REFMAC5 for the refinement of macromolecular crystal structures. *Acta Crystallogr. D Biol. Crystallogr.* **67**, 355-367 (2011).
9. Emsley, P. & Cowtan, K. Coot: model-building tools for molecular graphics. *Acta Crystallogr. D Biol. Crystallogr.* **60**, 2126-2132 (2004).
10. DeLano, W.L. The case for open-source software in drug discovery. *Drug Discov. Today* **10**, 213-217 (2005).
11. Gasteiger, E. *et al.* ExPASy: the proteomics server for in-depth protein knowledge and analysis. *Nucleic Acids Res.* **31**, 3784-3788 (2003).
12. Chen, Y.H., Yang, J.T. & Martinez, H.M. Determination of the secondary structures of proteins by circular dichroism and optical rotatory dispersion. *Biochemistry* **11**, 4120-31 (1972).
